# Imaging patterns and prognosis of proximal and distal small subcortical infarcts

**DOI:** 10.1007/s10072-025-08177-9

**Published:** 2025-04-15

**Authors:** Stefano Mombelli, Chiara Rinaldi, Vanessa Palumbo, Anna Poggesi, Patrizia Nencini, Gabriele Vandelli, Giorgio Busto, Rosario Pascarella, Enrico Fainardi, Maria Luisa Zedde, Francesco Arba

**Affiliations:** 1 NEUROFARBA Department, University of Florence, Careggi University Hospital, Florence, Italy; 2 Stroke Unit, Careggi University Hospital, Florence, Italy; 3 Neurology Unit, Stroke Unit, Azienda Unità Sanitaria Locale - IRCCS Di Reggio Emilia, Reggio Emilia, Italy; 4 Department of Experimental and Clinical Biomedical Sciences, Careggi University Hospital, Neuroradiology Unit, University of Florence, Florence, Italy; 5 Neuroradiology Unit, Ospedale Santa Maria della Misericordia, AULSS 5 Polesana, Rovigo, Italy; 6 Neurology Unit, Santo Stefano Hospital, Prato, Italy

**Keywords:** Stroke, Small Vessel Disease (SVD), Small Subcortical infarcts (SSI), Early Neurological Deterioration (END), Modified Rankin scale (mRS)

## Abstract

**Introduction:**

Small subcortical infarcts (SSI) are one of the hallmarks of cerebral small vessel disease and have been classified in two different imaging patterns: proximal-SSI (p-SSI) and distal-SSI (d-SSI), according to the shape, size and location to the parent artery. We aimed to investigate imaging and prognosis differences between p-SSI and d-SSI.

**Methods:**

We retrospectively reviewed records of consecutive patients with SSI admitted at two Stroke Units. We assessed location, shape and axial diameter of p-SSI and d-SSI with computed tomography (CT) and/or magnetic resonance (MR) imaging blinded to clinical data. Outcomes were: Early Neurological Deterioration (END), length of hospital stay, rehabilitation after discharge and functional status at 3 months assessed with modified Rankin Scale (mRS). We assessed independent associations between type of subcortical stroke and outcomes with logistic and ordinal regression analysis.

**Results:**

We included 292 patients, mean (+ -SD) age 67.42 (+ -12.41) years, 205 (70%) males, median (IQR) NIHSS = 4 (2–5); END occurred in 57 (20%) patients. Compared with d-SSI, p-SSI was associated with non-rounded shape (82%vs 65%,p = 0.005), lesion diameter > 15 mm (34% vs 10%,p < 0.001), infratentorial location (39% vs 23%,p = 0.005), absence of white matter changes (22% vs 12%,p = 0.035). END occurred more frequently in p-SSI (44% vs 10%,p < 0.001; OR = 7.23;95%CI = 3.73–14.03). In p-SSI, length of hospital stay was more frequently longer than six days (58% vs 40%,p = 0.005; OR = 1.78;95%CI = 1.04–3.07) and a shift towards worse mRS (cOR = 2.47;95%CI = 1.46–4.18) was observed.

**Conclusions:**

d-SSI and p-SSI have different imaging and prognostic characteristics that may suggest a distinct etiological origin and possibly different therapeutic approach.

**Supplementary Information:**

The online version contains supplementary material available at 10.1007/s10072-025-08177-9.

## Introduction

Ischemic stroke is the second leading cause of death and disability in the world. Among the ischemic subtypes, small subcortical infarcts (SSI) represent around a fifth of the total and have been linked to cerebral small vessel disease etiology (SVD) [1, 2]. SVD is a pathological process that encompasses a wide range of clinical, cognitive, neuroimaging and neuropathological findings where the arterioles, capillaries and venules are affected, with a resulting brain damage in the cerebral white and deep grey matter [3–5].

In the recent STandards for Reporting Vascular Changes on Neuroimaging 2 (STRIVE- 2) [6], acute SSI have been classified as one of the ischemic hallmarks of SVD [7]. SSI have been defined as recent ischemic lesions in the territory of one perforating arteriole with imaging features or clinical symptoms consistent with occurrence in the previous few weeks [8]. It is generally thought that up to 85% of SSI are due to intrinsic small vessel abnormalities and are associated with other SVD features whereas about 15% are caused by a perforating artery atheroma or embolism [6, 9]. Two different imaging patterns of SSI have been identified, based on imaging appearances, i.e. the proximal and distal SSI (p-SSI and d-SSI) [10]. Specifically, p-SSI refers to an infarct located adjacent to the parent artery, extending toward its basal surface and usually associated with parent artery disease, such as atherosclerosis, which can lead to the occlusion of a perforating artery's orifice while d-SSI refers to an infarct located farther from the parent artery, generally caused by small vessel pathology, such as lipohyalinosis or fibrinoid degeneration [9–11]. Although the functional outcome of SSI is generally fair, about 20–30% may experience early neurological deterioration (END) [12–14], with unclear mechanisms and risk factors. Furthermore, factors that may influence clinical outcomes have not been fully elucidated since several studies included mainly Asiatic population and is not clear whether results could be translated with the same magnitude of effect on other populations with different ethnicity.

In patients with ischemic stroke due to small subcortical infarcts, we aimed to investigate putative differences on imaging patterns and early and late prognostic differences between p-SSI and d-SSI.

## Materials and methods

### Patients

We retrospectively reviewed records of consecutive patients with acute ischemic stroke admitted at two Italian Stroke Units (Careggi University Hospital, Florence, between February 2009 and December 2022, and Azienda Unità Sanitaria Locale, Reggio Emilia, between December 2019 and December 2022). Inclusion criteria were: 1) clinical presentation of typical lacunar syndrome according to the Oxfordshire Community Stroke Project classification (OCSP) [15] or atypical presentation [16, 17]; 2) evidence of a small subcortical lesion attributable to a perforating arteriole in one of the following supratentorial and infratentorial vascular territory: lenticulostriate, basilar artery, posterior cerebral or posterior communicating artery; 3) ischemic lesion visible on computed tomography (CT) scan or on magnetic resonance (MR); 4) time between symptoms onset and hospital admission < 72 h. Our exclusion criteria consisted of patients with ischemic stroke with: 1) cortical lesions or multiple lesions; 2) determined etiology; 3) patients admitted after 72 h from symptoms onset. The study is compliant with the principles of the Declaration of Helsinki and was approved by each local institutional review board. Written informed consent was obtained from patients or legal representatives.

### Clinical data

We collected baseline demographic and clinical information for all study participants including age, sex, cardiovascular risk factors such as hypertension (defined by a previous use of any anti-ipertensive medication at home and/or systolic blood pressure > 140 mmHg or diastolic blood pressure > 90 mmHg at admission), diabetes mellitus (defined by a previous use of glucose-lowering medication at home or blood glucose level ≥ 126 mg/dl at fast or ≥ 200 mg/dl at one random measurement), hyperlipidemia (previous use of lipid-lowering medication or fasting total cholesterol > 200 mg/dL during hospitalization), alcoholic use, habitual smoking (current smoking), history of any cardio-cerebrovascular disease such as ischemic cardiopathy, peripheral artery disease, atrial fibrillation and history of previous Stroke/Transient Ischemic Attack (TIA), current home medication (antiplatelets, anticoagulants, antihypertensive, lipid-lowering), clinical data at admission and during hospitalization. The following lacunar syndromes were considered as per OCSP classification [15]: pure motor hemiparesis, pure sensory syndrome, sensorimotor syndrome, ataxic-hemiparesis, dysarthria-clumsy hand. Furthermore, we included patients with atypical presentations (i.e., non-lacunar syndromes) and evidence of an ischemic lesion consistent with SSI. Atypical presentations refer to symptoms that do not fit with the classic lacunar syndromes as defined by the OCSP classification [16, 17]. Stroke severity was measured with the National Institute of Health Stroke Scale (NIHSS) by a stroke neurologist. Laboratory data were recorded. Discharge destinations were classified as follows: at home without rehabilitation, rehabilitation at home, rehabilitation centre. All patients underwent at least a 12-lead ECG and Echocardiography to find signs of hypertensive cardiopathy (hypertrophy or altered diastolic relaxation of the left ventricle) and excluded structural heart diseases.

### Imaging data

**Fig. 1:**
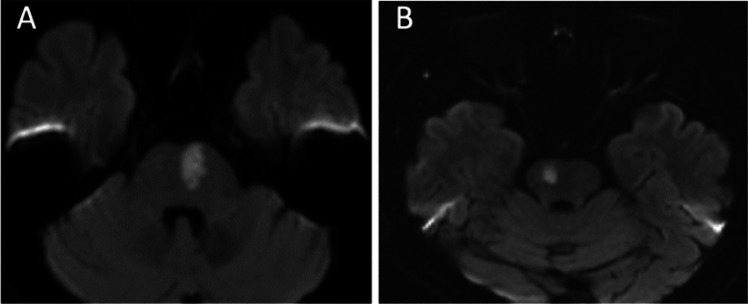
Ischemic lesions on MR with infratentorial p-SSI (A) and d-SSI (B) pattern

**Fig. 2:**
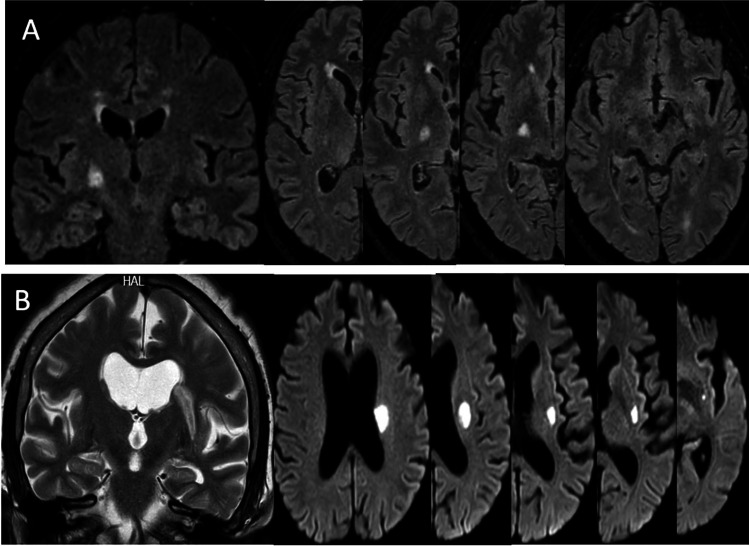
Ischemic lesions on MR with supratentorial d-SSI (A) and p-SSI (B) pattern on axial and coronal sections

**Fig. 3:**
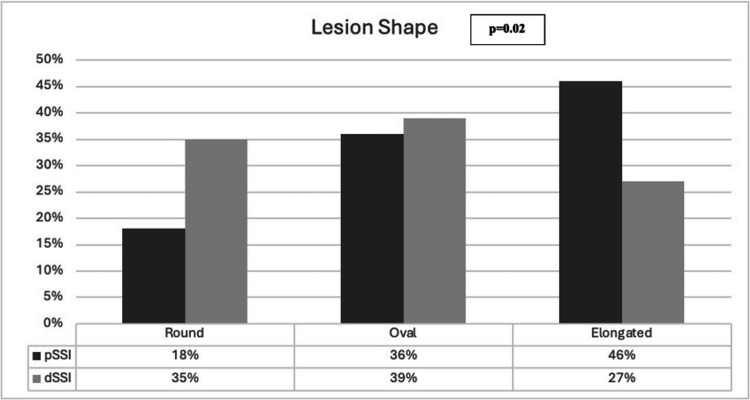
Lesion shape on imaging of patients with p-SSI and d-SSI. p-SSI = single proximal subcortical infarction; d-SSI = single distal subcortical infarction

All patients underwent a brain CT at admission and a follow-up CT after 24–36 h. In case of undetectable lesion at CT or other clinical judgement of the stroke physician, MR scan (1.5 Tesla) was performed. For patients who underwent MR scan, a minimum standard of sequences including DWI, FLAIR, T1-weighted imaging, T2-weighted imaging and T2* were obtained. All images were retrospectively reviewed by stroke neurologists or a neuroradiologist blinded to clinical information. On the visible (either on CT or MR) lesion, we assessed: 1) SSI dimension on the axial plane on CT, by measuring axial diameter and longitudinal extent of the lesion, estimated by counting the number of slices in which the lesion was visible (e.g. for CT with slice thickness of 5 mm, a lesion spanning three slices was estimated to have a longitudinal diameter of 15 mm; and for slice thickness of 2 mm a lesion spanning of eight slices was estimated to have a longitudinal diameter of 16 mm); 2) lesion pattern, defined as p-SSI if the ischemic lesion was located close to the presumed parent artery (e.g. extending from the basal surface of the middle cerebral artery), and d-SSI if the lesion was located in the distal portion of the perforating arteries arising from the presumed parent artery (Fig. 1 and 2*)*; 3) lesion shape, defined as round if major and minor axis had similar length, oval if major axis was longer than minor axis and elongated if major axis was at least twice as long as minor axis, 4) lesion location (e.g. supratentorial or infratentorial); 5) SVD markers: white matter changes (WMC), assessed by Van Swieten Scale (VSS), presence of old infarcts (lacunar vs non-lacunar). All patients underwent assessment of the cervico-cephalic arteries via ultrasound of the supra-aortic trunks/transcranial doppler or CT angiography/MR angiography. Intracranial stenosis was defined as a reduction of the vessel lumen > 50% due to atherosclerotic process and identified with CT angiography/MR angiography.

### Outcomes

We considered the following functional outcomes: early neurological deterioration (END), defined as worsening of at least two points at NIHSS or at least one point in the motor item (4, 5a, 5b, 6a, 6b) within the first 72 h from symptom onset, length of hospital stay >  = 6 days, discharge with rehabilitation facilities, functional independency three months after the index stroke measured with the modified Rankin Scale (mRS).

### Statistical analysis

The general characteristics of the population were described with a statistics summary. Categorical data were expressed as raw numbers (N,%). For continuous variables we performed Kolmogorov–Smirnov Test for normality and expressed data accordingly. To analyse group differences between categorical variables we used Pearson's χ2. To analyse the differences between variables we used the Mann–Whitney test. We assessed independent associations between type of subcortical stroke (p-SSI and d-SSI) and outcomes with logistic regression adjusting for age, sex, baseline NIHSS, tissue plasminogen activator (rt-PA) administration, hypertension and diabetes. We expressed the association with odds ratio (OR) and 95% confidence interval (95% CI). Differences were considered statistically significant at a level of p < 0.05 for a two-sided test for independent associations. Statistical analysis was performed with SPSS for Mac software (version 26.0; SPSS, ArmonkIBM Corp.).

## Results

### Baseline characteristics

In the study period, a total of 308 patients were diagnosed as having a SSI, of which 16 (5%) did not have either CT and MR detectable imaging lesion. This left 292 patients for the final analysis, of which 85 (29%) had p-SSI and 207 (71%) had d-SSI. Table 1 shows baseline characteristics of the study population. Median (IQR) age was 68 (58–77) years, 205 (70%) were males, median (IQR) NIHSS was 4 (2–5), 230 (80%) presented with a lacunar syndrome according to OCSP classification; 67 (23%) patients received rt-PA, 253 (87%) had hypertension and 81 (28%) had diabetes. In 123 (42%) SSI was visible on CT whereas in 169 (58%) SSI was visible only after MR. Compared with d-SSI, patients with p-SSI had higher median NIHSS (4 vs 3; p = 0.002), had more frequently intracranial atherosclerosis (39% vs 16%; p < 0.001), received more frequently rt-PA (31% vs 20%, p = 0.047) and less frequently antihypertensive drugs at home (42% vs 59%; p = 0.012). There were no other differences regarding anamnestic and clinical factors.

**Table 1:** Baseline characteristics of patients with p-SSI and d-SSI

	**Total** **n = 292**	**p-SSI** **n = 85**	**d-SSI** **n = 207**	**p**
Age, years, median, (IQR)	68(58–77)	68(58–76)	68(58–77)	0.676
Sex, male	205 (70)	59 (70)	146 (71)	0.960
Hypertension	253 (87)	73 (86)	180 (87)	0.806
Diabetes Mellitus	81 (28)	22 (26)	59 (29)	0.650
Hyperlipidemia	206 (71)	56 (66)	150 (73)	0.262
Atrial Fibrillation	6 (2)	2 (2)	4 (2)	0.818
Ischemic Cardiopathy	20 (7)	4 (5)	16 (8)	0.353
Hypertensive Cardiopathy	155 (55)	49 (59)	106 (53)	0.312
Previous Stroke/TIA	51 (18)	11 (13)	40 (20)	0.192
Intracranial atherosclerosis	65 (22)	32 (39)	33 (16)	< 0.001
Carotid Lesion	21 (7)	3 (4)	18 (9)	0.453
Peripheral Artery Disease	20 (7)	5 (6)	15 (7)	0.675
Patent Foramen Ovale	7 (14)	3 (14)	4 (14)	1.000
Smoking	131 (46)	32 (39)	99 (48)	0.133
Alcohol	54 (19)	17 (20)	37 (18)	0.671
Systolic BP, mmHg, median, (IQR)	163(146–182)	167(150–190)	160(145–180)	0.068
Diastolic BP, mmHg, median, (IQR)	88(80–100)	88(80–100)	88(80–100)	0.745
Baseline glucose, mg/dL, median, (IQR)	111(97–138)	109(96–137)	111(98–138)	0.964
Admission NIHSS, median, (IQR)	4 (2–5)	4 (3–7)	3 (2–5)	0.002
rt-PA	67 (23)	26 (31)	41 (20)	0.047
OCSP classification				0.362
LACS	230 (80)	69 (82)	161 (79)	
TACS	1 (-)	0 (-)	1 (-)	
PACS	13 (5)	6 (7)	7 (3)	
POCS	44 (15)	9 (11)	35 (17)	
Antiplatelet/s	80 (28)	20 (24)	60 (29)	0.370
Anticoagulant/s	10 (3)	2 (2)	8 (4)	0.883
Antihypertensive/s	157 (54)	36 (42)	121 (59)	0.012
Lipid-lowering medication/s	65 (22)	13 (15)	52 (25)	0.067
CT scan	123 (42)	42 (49)	81 (39)	0.106

All values are to be considered as N(%) unless otherwise specified; p-SSI = single proximal subcortical infarction; d-SSI = single distal subcortical infarction; SD = Standard Deviation; IQR = interquartile range; BP: Blood Pressure; TIA = Transient Ischemic Attack; NIHSS = National Institute of Health Stroke Scale; OCSP = Oxfordshire Community Stroke Project; LACS = lacunar syndrome; TACS = total anterior circulation syndrome; PACS = partial anterior circulation syndrome; POCS = posterior circulation syndrome; CT = computed tomography; MR = magnetic resonance imaging; rTPA = tissue plasminogen activator

### Radiological characteristics

Regarding the radiological characteristics (Table 2), 247 (85%) patients had some extent of white matter changes on CT/MR, median (IQR) axial diameter of the ischemic lesion was 11.00 (6.50–14.00) mm and lesion location was infratentorial in 80 (27%) patients. Patients with p-SSI had a median diameter lesion of 15.00 (11.00–19.00) mm compared with 10.00 (0.90–13.00) mm of d-SSI (p < 0.001), extended more than 3 slices (52% vs 8%, p < 0.001) and were more frequently infratentorial (39% vs 23%, p < 0.005). White matter changes were more frequent in patients with d-SSI (88% vs 78%, p < 0.037). The morphology of the lesion in axial sections was non-round in 201 (70%) patients (82% p-SSI patients vs 65% d-SSI p = 0.005). Elongated shape was more frequent in p-SSI patients (46% vs 27%) while rounded shape prevailed in d-SSI (35% vs 18%) (Fig. 3), and oval shape was similar between the two groups (36% in p-SSI vs 39% in d-SSI).

**Table 2:** Imaging characteristics of patients with p-SSI and d-SSI

	**Total** **(n = 292)**	**p-SSI** **(n = 85)**	**d-SSI** **(n = 207)**	**p value**
White Matter Changes	247 (85)	65 (78)	182 (88)	0.037
Previous lacunar infarct	110 (38)	31 (37)	79 (38)	0.841
Previous non lacunar infarct	19 (7)	8 (10)	11 (5)	0.266
Axial diameter, mm, median, (IQR)	11.00 (6.50–14.00)	15.00 (11.00–19.00)	10.00 (0.90–13.00)	< 0.001
Diameter > 15 mm	227 (79)	78 (93)	149 (73)	< 0.001
> 3 Slices	60 (21)	43 (52)	17 (8)	< 0.001
Infratentorial location	80 (27)	33 (39)	47 (23)	0.005
Non-round shape	201 (70)	68 (82)	133 (65)	0.005

All values are to be considered as N(%) unless otherwise specified; p-SSI = single proximal subcortical infarction; d-SSI = single distal subcortical infarction; SD = Standard Deviation

### Outcomes

In the general population of 292 patients, 57 (20%) developed END (Table 3), 141 (45%) had length of hospital stay > 6 days, 150 (52%) required rehabilitation program at discharge and 193 (76%) were functionally independent (mRS 0–2) at 90 days. Compared with d-SSI, p-SSI patients had more frequently END (44% vs 10%, p < 0.001), a length of hospital stay > 6 days (58% vs 40%, p = 0.005) and required more frequently a post discharge rehabilitation (70% vs 45%, p < 0.001) while d-SSI patients were more functionally independent (mRS 0–2) at 90 days (84% vs 55%, p < 0.001). P-SSI pattern was independently associated with a seven-fold increase in the odds of END (OR = 7.23, 95% CI = 3.73–14.02), increased length of hospital stay > 6 days (OR = 1.79, 95% CI = 1.04–3.07) and rehabilitation program (OR = 2.41, 95% CI = 1.35–4.31) and with a less favourable functional outcome (mRS 0–2) at 90 days (OR = 0.27, 95% CI = 0.13–0.54).

**Table 3:** Outcomes of patients with p-SSI and d-SSI

	**Total** **(n = 292)**	**p-SSI** **(n = 85)**	**d-SSI** **(n = 207)**	**p**	**Adj OR** **(95% IC)**
END	57 (20)	37 (44)	20 (10)	< 0.001	OR = 7.23 (3.73–14.02)
Length of Hospital stay > = 6 days	141 (45)	49 (58)	82 (40)	0.005	OR = 1.79 (1.04–3.07)
Rehabilitation	150 (52)	58 (70)	92 (45)	< 0.001	OR = 2.41 (1.35–4.31)
mRS at 90 days 0–2	193 (76)	41 (55)	152 (84)	< 0.001	OR = 0.27 (0.13–0.54)

The odds ratios come from logistic regression adjusted for age, sex, baseline NIHSS, rtPA, hypertension, diabetes. p-SSI = single proximal subcortical infarction; d-SSI = single distal subcortical infarction; OR = odds ratio; CI = Confidence Interval; END = early neurological deterioration; mRS = modified Rankin Scale; rtPA = tissue plasminogen activator

## Discussion

In this study we investigated the clinical and imaging patterns, as well as the prognostic courses of p-SSI and d-SSI. We found higher stroke severity and more frequent intracranial atherosclerosis in p-SSI, whereas hypertension was more frequent in d-SSI. Regarding imaging characteristics, p-SSI had larger lesion size and different morphology and topography and were less associated with pre-existing white matter changes compared with d-SSI. Such differences suggest that p-SSI and d-SSI, although both located in a perforating artery territory, may be two distinct pathological processes. Moreover, we observed that p-SSI patients had more frequently END, longer hospital stay, required more frequently rehabilitation facilities and were less likely independent three months after the index stroke, suggesting that p-SSI and d-SSI have different prognostic implications.

Although SSI are usually regarded as milder strokes subtype, they have been associated with cognitive impairment [18], recurrent cerebrovascular events and long-term sequelae [19]. Since the first descriptions of branch atheromatous disease (BAD) [9], proximal and distal SSI patterns have been identified. Prognostic implications of these two lesion patterns have been explored mainly on Asiatic population [20, 21] and need external validation. In a recent work conducted by Nam et al. [22] p-SSI was associated with END and authors identified plaque instability and embolization from parent artery atheroma as key mechanisms of END. However, we should specify that intracranial atherosclerosis has a higher prevalence in Asiatic population compared to other ethnic groups. In keeping with this study, we observed that nearly a half of patients with p-SSI had END, possibly alluding to the greater size of the ischemic lesion due to the proximal site of SSI [23] and the consequent higher neurological deficit and risk of lesion expansion [13, 24, 25].

Previous studies suggested that CT perfusion patterns may predict END, however, conclusive evidence is lacking [26]. END is associated with disability in the long term [12], and our study is in line with this hypothesis, showing less likely functional independency at three months. We also observed longer hospital stay, twice the risk of rehabilitation programs in patients with p-SSI. Such results are in keeping with a similar study on the Asiatic population, which found longer hospital stay and worse functional status at 12 months in patients with p-SSI patients [27]. Lesion topography and morphology may explain the different outcomes between p-SSI and d-SSI. P-SSI were more frequently infratentorial and had an elongated shape in approximately half of cases. The infratentorial location may be responsible for the higher stroke severity of p-SSI and the subsequent need of rehabilitation facilities, given that the ostium of the perforating artery is located ventrally to the brainstem, close to the course of the pyramidal tracts [28–30] while the elongated shape is the result of the presumed occlusion of the proximal portion of a perforating artery [10]. Consistently with the latter, supratentorial p-SSI patients had a larger axial diameter and a lesion longer than three slices on axial imaging sequences (i.e. elongated shape in coronal sequences)*,* similar to previous data, whereas infratentorial p-SSI patients had a larger axial diameter in infratentorial SSI [31]. Larger lesion burden and proximity to corticospinal tracts involving motor pathways are consistent with higher stroke severity and the observed worse outcomes. Besides different acute ischemic lesion patterns, we also observed a difference in pre-existing markers of small vessel disease, since white matter changes were more frequent in patients with d-SSI, alluding to a common pathogenetic mechanism involving the small vessels in the brain (e.g. lypohyalinosis) and supporting a different process for the small vessel involved in p-SSI, in agreement with previous studies [10, 22]. Accordingly, in p-SSI we observed a higher rate of intracranial atherosclerosis of the patent artery, suggesting an atheroma at the origin of the perforating artery, rather than a pathological process of the vessel wall such as lypohyalinosis, as hypothesized in d-SSI [32, 33]. It should be noted that the acute ischemic lesion with CT scan was visible in less than a half of patients, whereas in the remaining cases MR was necessary to visualize the lesion. Although p-SSI were more frequently detectable with CT scan, more than a half of cases needed MR, confirming the better diagnostic accuracy of MR [34].

Our results point out that the distinction between p-SSI and d-SSI is relevant since the two patterns have distinct early and long-term outcomes. Early identification of SSI pattern is therefore important to manage unfavourable outcomes, above all END. Furthermore, early identification of SSI pattern may help implementation of therapeutic strategies to reduce the burden of the disease and healthcare costs: we found that a half of SSI needed rehabilitation facilities and the odds were doubled in patients with p-SSI, thus increasing healthcare costs and highlighting the unmet need of preventing strategies to tackle unfavourable outcomes in such patients [35]. Studies with larger sample sizes could identify more precisely predictive factors and attempt to build a probabilistic score for the early clinical identification of p-SSI. Whether clinical or further imaging features may help early identification of patients at risk of END remains to be defined.

Our study had limitations. The retrospective design from medical records may generate an assessment bias, since there was not a pre-specified collection form or study protocol. However, the results are biologically plausible and are in keeping with the existing literature. A selection bias is also possible, since the data were from patients admitted to two Stroke Units and we lack data from patients admitted in other wards or in intensive care unit. On the other hand, this may reassure about the quality of data since patients were managed with adherence to protocols and homogeneous clinical practice. Another limit was the lack of an operational definition of p-SSI without dimensional cut-off. However, STRIVE-II criteria [6] suggested that SSI not due to intrinsic small vessel disease had different size and shape without a defined dimensional criterion. Moreover, a previous analysis from a large trial on lacunar infarcts showed that in patient with SSI there was a relevant variability in shape and volume [36]. We acknowledge that our inclusion criteria could lend to imprecision in selection of patients, however, we decided to be more inclusive given the evolving concept and definition of SSI. Again, there was not a pre-specified timing for brain imaging nor neuroimaging protocol, and this could introduce a detection bias in assessing lesion morphology. However, all patients included received at least two brain imaging scans within 36 h from admission, and we demonstrated that CT could detect the ischemic lesion in approximately half of cases, meaning that in the remaining cases MR is necessary. We recognize that our approach was pragmatic, and that diagnosis of the acute ischemic lesion was performed with two different techniques, but our results could be easily translated into clinical practice.

## Conclusion

Our study confirms that there are two distinct subtypes of small subcortical infarcts, with different prognosis and likely different pathogenetic mechanisms. Around in a half of cases MR is necessary to demonstrate the SSI subtype. Early neurological deterioration occurred more frequently in p-SSI, underlying the need of rapid identification and management of the SSI subtype. Similarly, in p-SSI patients hospital stay was longer, rehabilitation program was more frequent and functional independency was less likely. Further studies are necessary for a better understanding of the underlying pathogenetic mechanisms and future clinical trials aimed at preventing early neurological deterioration are warranted.

## Supplementary Information

Below is the link to the electronic supplementary material.

Supplementary file1 (DOCX 22 KB)

## Data Availability

Data are available upon reasonable request at Azienda Ospedaliero Universitaria Careggi, Firenze.
