# Supplementary material for: Imaging patterns and prognosis of proximal and distal small subcortical infarcts
Source: Neurol Sci. 2025 Apr 15;46(8):3709–17. doi: 10.1007/s10072-025-08177-9 (PMC12267300; doi:10.1007/s10072-025-08177-9)
Supplement: Supplementary file 1 — Supplementary file1 (DOCX 22 KB) [file 10072_2025_8177_MOESM1_ESM.docx]

**Supplementary Materials**

**Table 1 Multivariable Logistic Regression for relative factors associated with Early Neurological Deterioration**

|  | p | aOR | 95% CI |
| --- | --- | --- | --- |
| Age | 0.285 | 1.02 | 0.99-1.04 |
| Sex | 0.182 | 1.67 | 0.79-3.56 |
| NIHSS at admission | 0.551 | 0.97 | 0.87-1.08 |
| Rt-PA | 0.061 | 2.05 | 0.97-4.35 |
| P-SSI | <0.001 | 7.23 | 3.27-14.04 |
| Hypertension | 0.612 | 0.78 | 0.30-2.03 |
| Diabetes | 0.785 | 1.11 | 0.53-2.29 |

Hosmer-Lemeshow Test: 0.82

aOR=adjusted Odds Ratio; CI= Confidence Interval; NIHSS=National Institutes of Health Stroke Scale; rt-PA=tissue plasminogen activator p-SSI= single proximal subcortical infarction.

**Table 2 Multivariable Logistic Regression for relative factors associated with Lenght of hospital stay ≥ 6 days**

|  | p | aOR | 95% CI |
| --- | --- | --- | --- |
| Age | 0.626 | 1.01 | 0.99-1.03 |
| Sex | 0.018 | 0.52 | 0.30-0.90 |
| NIHSS at admission | 0.017 | 1.12 | 1.02-1.24 |
| Rt-PA | 0.145 | 1.57 | 0.86-2.89 |
| P-SSI | 0.032 | 1.81 | 1.05-3.11 |
| Hypertension | 0.862 | 0.94 | 0.45-1.95 |
| Diabetes | 0.315 | 1.33 | 0.76-2.31 |

Hosmer-Lemeshow Test: 0.24

aOR=adjusted Odds Ratio; CI= Confidence Interval; NIHSS=National Institutes of Health Stroke Scale; rt-PA=tissue plasminogen activator p-SSI= single proximal subcortical infarction.

**Table 3 Multivariable Logistic Regression for Relative Factors Associated with Rehabilitation**

|  | p | aOR | 95% CI |
| --- | --- | --- | --- |
| Age | 0.102 | 1.02 | 0.99-1.04 |
| Sex | 0.951 | 0.98 | 0.56-1.72 |
| NIHSS at admission | <0.001 | 1.30 | 1.15-1.47 |
| Rt-PA | 0.257 | 0.69 | 0.36-1.32 |
| P-SSI | 0.003 | 2.45 | 1.37-4.40 |
| Hypertension | 0.364 | 1.41 | 0.67-2.98 |
| Diabetes | 0.346 | 1.32 | 0.74-2.36 |

Hosmer-Lemeshow Test: 0.56

aOR=adjusted Odds Ratio; CI= Confidence Interval; NIHSS=National Institutes of Health Stroke Scale; rt-PA=tissue plasminogen activator p-SSI= single proximal subcortical infarction.

**Table 4 Multivariable Logistic Regression for Relative Factors Associated with mRS 0-2 at 90 days**

|  | p | aOR | 95% CI |
| --- | --- | --- | --- |
| Age | <0.001 | 0.94 | 0.91-0.97 |
| Sex | 0.491 | 1.30 | 0.62-2.71 |
| NIHSS at admission | <0.001 | 0.81 | 0.71-0.91 |
| Rt-PA | 0.116 | 0.52 | 0.23-1.17 |
| P-SSI | <0.001 | 0.23 | 0.11-0.47 |
| Hypertension | 0.047 | 0.23 | 0.54-0.98 |
| Diabetes | 0.055 | 0.49 | 0.24-1.02 |

Hosmer-Lemeshow Test: 0.07

aOR=adjusted Odds Ratio; CI= Confidence Interval; NIHSS=National Institutes of Health Stroke Scale; rt-PA=tissue plasminogen activator p-SSI= single proximal subcortical infarction.
